# SARS-CoV-2 Infection Anxiety, Knowledge and Attitudes in University Degree Pregnant Women from Romania—A Cross-Sectional Observational Survey in the First Two Pandemic Years

**DOI:** 10.3390/vaccines11010035

**Published:** 2022-12-23

**Authors:** Madalina Preda, Rares Sebastian Dinu, Irina Prasacu, Loredana Sabina Cornelia Manolescu

**Affiliations:** 1 Department of Microbiology, Parasitology and Virology, Faculty of Midwives and Nursing, “Carol Davila” University of Medicine and Pharmacy, 020021 Bucharest, Romania; 2 Research Department, Marius Nasta Institute of Pneumology, 050159 Bucharest, Romania; 3 Prahova County Ambulance Service, 100461 Ploiești, Romania; 4 Department of Fundamental Sciences, Faculty of Pharmacy, “Carol Davila” University of Medicine and Pharmacy, 050474 Bucharest, Romania; 5 Department of Virology, Institute of Virology “Stefan S. Nicolau”, 030304 Bucharest, Romania

**Keywords:** pregnancy, coronavirus, COVID-19, KAP, vaccines, immunization

## Abstract

The severe acute respiratory syndrome coronavirus 2 (SARS-CoV-2) pandemic had a high economic cost, morbidity, and death toll. Due to high rates of mortality and morbidity from coronavirus disease 2019 (COVID-19), pregnant women were at particular risk during this pandemic. We designed and conducted a cross-sectional observational survey in Romanian pregnant women to evaluate the outcome of the SARS-CoV-2 pandemic along with the preventive measures taken by authorities. We applied a 43-item questionnaire. We included 147 women over 18 years old from Romania who were pregnant or gave birth between March 2020 and March 2022. All pregnancies were monitored, most of them by a gynecologist, and only 23% faced rescheduled pregnancy visits. The majority (84%) were screened through the TORCH panel, 95.91% felt anxious because of the pandemic, 87% followed SARS-CoV2 preventive recommendations, and 82% were not infected with the coronavirus. Additionally, 80% were vaccinated against COVID-19. They felt the consequences of the pandemic through anxiety, and their level of anxiety influenced their vaccination decisions, the TORCH testing and the method of giving birth despite the level of education. Fortunately, their pregnancies were monitored properly, and there were no consequences noticed for fetuses at birth.

## 1. Introduction

The world has recently experienced several challenges such as climate change, natural resources depletion, COVID-19, and the Russian–Ukrainian war; all of these have been in our thoughts and have changed our behavior [1].

Out of all these challenges, the COVID-19 pandemic that was triggered by the emergence of the severe acute respiratory syndrome coronavirus 2 (SARS-CoV-2) [2] was well documented by the medical world. The COVID-19 severity spectrum spans from mild or moderate to severe or critical illness that can be fatal [3].

COVID-19 symptoms might have a wide range of manifestation. In the most vulnerable patients, viral pneumonia’s initial signs and symptoms can swiftly advance to severe metabolic acidosis, acute respiratory distress syndrome (ARDS), sepsis, septic shock, abrupt cardiac damage, and coagulopathy [3]. Additionally, treatment options may vary according to the severity, from no medication necessary to severe evolution of the disease, with the need for pulmonary rehabilitation [4].

Due to their high risk of morbidity and mortality from COVID-19, pregnant women are particularly at risk during the current COVID-19 pandemic [2]. When compared to non-pregnant women with COVID-19, pregnant women are typically at a higher risk of serious disease, hospitalization, invasive mechanical ventilation, admission to the intensive care unit, preeclampsia, and mortality [2]. Upon implantation of the embryo, the female immune system undergoes huge changes which, while remitting some diseases, enhances the risk of an inflammatory response to common infections, of which COVID-19 is one. Pregnant women with COVID-19 are also more likely to experience unfavorable birth outcomes, such as premature births, abortion, cesarean delivery, and neonatal ICU admissions, making neonatal morbidity and mortality more likely than in pregnant women without COVID-19 [2]. Pregnant women have a more severe case of SARS-CoV-2 infection than non-pregnant women, which increases the risk of hospital admission, intensive care unit admissions, and death [3].

Another important influence of COVID-19 is the anxiety induced in pregnant women, not only by the possibility of infection, but also through other aspects, such as isolation from the newborn in the case of SARS-CoV-2 infection in the mother, and reduced contact with loved ones during admission in the healthcare facility [5]. Additionally, restraint tactics have had an impact on a range of lifestyle practices, with consequences for mental health, including social interactions, mobility or changes in physical activity, alcohol usage, eating patterns, smoking, and sleeping [5]. The World Health Organization reported that 10% of pregnant women and 13% of postpartum women had a mental health condition prior to the COVID-19 pandemic [6].

For COVID-19, the possibility of prevention through vaccination was rapidly available, even in 2020, throughout the world and in Romania as well [7,8,9]. The vaccination had a favorable effect upon pregnant women.

There are several reasons why vaccination is important. For example, in an Ethiopian study, only a fraction (2.2%) of all samples were fully immunized, with 91 (14.4%; 95%CI: 11.7–17.3%) of the responders having received at least one dose of the COVID-19 vaccine [2]. Of individuals who received the vaccine, the majority (59, 64.8%) reported no post-vaccination symptoms, while 32 (35.2%) reported minor adverse effects, but vaccine safety concerns due to the worry of negative side effects for the fetus or the mother were the most common reasons for refusing the COVID-19 vaccination [2]. One of the most crucial strategies for assisting a child’s health and survival, particularly during the COVID-19 epidemic, is to breastfeed them for six months exclusively [10]. An impediment to this could be the isolation of newborns from SARS-CoV-2 positive mothers.

Concerning inflammatory changes, elevated C-reactive protein levels and lymphocytopenia were frequent [11]. In a review of the placenta affected by SARS-CoV2 infection of the mother, lesions were found in the placentas of women with COVID-19 infections. However, the degree of the lesions was not compared between the two groups of women—infected and not infected [12]. Very rarely, SARS-CoV-2 can be found in placental tissue when maternal macrophages carry the virus, which attaches to and kills the villous trophoblast, causing acute and persistent intervillositis similar to some other placental infections [13]. Other studies on fetal death noticed strong placental inflammatory responses, including villitis and intervillitis, observed in all cases, raising the hypothesis that SARS-CoV-2 had an immediate impact on the placenta [14]. This theory is supported by the discovery of SARS-CoV-2 in two placental and one amniotic fluid samples. Only one fetus was provided for autopsy, showing symptoms of illness [14].

Local government institutions and the WHO issued numerous recommendations to assist nations in maintaining essential health services during the COVID-19 pandemic because a deeper understanding of this new human challenge may still be lacking at this time [15]. In the context of the COVID-19 pandemic, knowledge advances intervention efforts and encourages the public’s preventive actions by acting as a crucial predictor of attitudes and behaviors [15]. To prevent and control diseases, it is vital to have the right information, attitude, and behaviors, since it is common knowledge that unclear understanding and unfavorable attitudes can lead to unwarranted fear and anxiety regarding developing communicable illnesses [15,16]. Mothers’ knowledge, attitudes, and behaviors are crucial for preventing and managing diseases, particularly contagious ones.

Few studies, to our knowledge, have examined the general public’s understanding of this new coronavirus and knowledge of its control in the Romanian pregnant women community. This study proposes to examine the outcome of the SARS-CoV-2 pandemic along with and the preventive measures taken by Romanian authorities in pregnant women with university degrees, that is, women who have access to all available knowledge from the field. To achieve this aim we evaluated the knowledge, emotional state—measured by anxiety level—attitudes, practices and possible influence on pregnancy monitoring in the context of the application of preventive measures against the spread of the SARS-CoV-2 virus. Thankfully, in this small sample there were no adverse outcomes.

## 2. Materials and Methods

### 2.1. Study Design, Setting, and Participants

We designed and conducted a cross-sectional observational survey to evaluate the impact that the SARS-CoV-2 pandemic and the preventive measures had on pregnant women. For an accurate design, we used the “Strengthening the Reporting of Observational Studies in Epidemiology” (STROBE) rules [17]. The STROBE checklist is added in the Supplementary Materials (File S1). The survey was conducted with the aid of medical students and graduates of the “Carol Davila” University of Medicine and Pharmacy from Bucharest in Romania, in one section of a University Hospital of Obstetric Gynecology from Romania. During the studied period, 1288 pregnant women presented to give birth. The volunteers helped the pregnant women to understand the questionnaire and gather accurate answers. The inclusion criteria for the participants were to be a pregnant woman at least 18 years old and to have Romanian citizenship. We included pregnant women or women who had given birth while applying various preventive measures against the spread of the SARS-CoV-2 virus between March 2020 and March 2022. We excluded participants under 18 years old, women who were unable to read and understand the questions from the questionnaire, incomplete questionnaires, pregnant women with severe physical conditions (severe hypertension, diabetes mellitus, cardiac disease, kidney or liver disease), severe pregnancy-related conditions (for example antepartum hemorrhage, pre-eclampsia, hyperemesis gravidarum, premature rupture of membranes), and/or severe mental conditions. Because of these exclusion criteria, our study group included women that were able to understand the events they were part of.

### 2.2. Survey Questionnaire and Data Collection

We used a 43-item questionnaire (Supplementary Materials file S2) with closed answers, which aimed to quantify various indicators for the data collection, and signed informed consent was given by each pregnant woman. The questionnaire was based on questionnaires already validated through publication [7,8] and approved by the Ethics Committee of the Obstetrics and Gynecology Hospital.

The questionnaire includes nine sections that aim to analyze the changes of behavior at the population level as a repercussion of the lockdown period, the impact of preventive measures against the spread of the SARS-CoV-2 virus had on the mental health of pregnant women, the economic impact of the lockdown period and preventive measures, and pregnancy monitoring and behavioral changes during the period of lockdown. The data collection during the study was carried out using the option Forms of the Google platform and the Google spreadsheet to collect and centralize the answers. All questions were mandatory, and they could only submit the questionnaire after answering them. Before replying to the questions, we informed each participant about the importance of the study and the way their answers would be used. The questionnaire’s introduction page included a section on participant consent after describing the study’s goals, purpose, and anticipated time frame. Each filled-in questionnaire was anonymized and given a number before analysis.

### 2.3. Ethical Approval

The questionnaire was peer-validated and approved by the Ethics Committee of the Obstetrics and Gynecology Hospital, Ploiești, Romania (41482/9 August 2022); all the procedures in the survey respected the ethical standards of the Helsinki Declaration. Informed consent was compulsory.

### 2.4. Statistical Analysis

The data from the questionnaire were analyzed by means of the Microsoft Office package Excel and IBM^®^ SPSS^®^ Statistics Version 23.0 software. For data processing, the COUNTIFS function in Excel was used to filter and sort the initial database. The anxiety generated by the pandemic and the fear of being infected with the pandemic virus was considered the most important variable and was, hence, considered as the main dependent variable for consecutive analysis. We considered one as the lowest level of anxiety and five as the highest level. Applied tests were descriptive ones. Since all data, except for age, were discrete variables, we also applied the non-parametric Chi test and Fisher’s Exact test. For measuring the magnitude of the effect for the comparisons we used an Odds Ratio (OR). For all tests, the threshold for statistical significance was considered to be 0.05.

## 3. Results

We included 147 women over 18 years old from Romania who were pregnant or gave birth between March 2020 and March 2022. General characteristics are included in Table 1.

Regarding the age distribution, the predominance of people aged between 18 and 35 can be observed, representing 69.4% (102 participants) of the study sample. Regarding occupation, 95.2% (140 participants) of mothers were employed, while the rest were students 2.7% (four participants) and households 2% (three participants). The marital status of the participants in the study was predominantly married (86.4% (127 participants)), with 12.9% (19 participants) being single, and only one person divorced.

From the point of view of the living environment, 140 of them lived in the urban environment, representing 95.2% of the study group, and seven lived in rural areas, representing 4.8% of the study group.

Depending on the level of education, the study participants were divided as follows: 143 of the mothers had university degrees (97.3% of the respondents), and four people had completed post-secondary school, representing 2.7% of the sample.

It can be seen that, from the point of view of the family situation, in 97.3% of the cases the child will be raised by a two-parent family (143 people from the study sample), while 2.7% will have a single-parent family (four people).

The percentage of pregnant women monitored during pregnancy was 100%.

Regarding the specialist who performed the pregnancy monitoring, 146 of them had their pregnancy monitored by a gynecologist; only one person had the pregnancy supervised by a midwife, while none of the respondents had their pregnancy monitored by a general practitioner.

**Figure 1:**
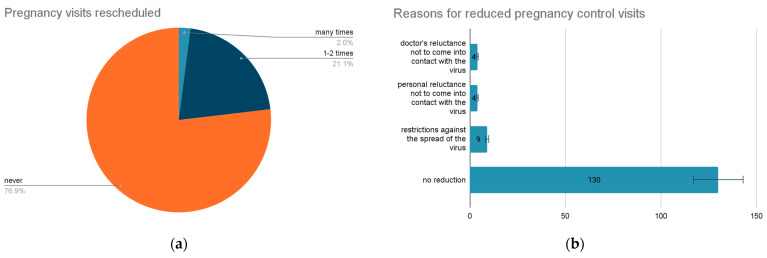
Pregnancy monitoring impacted by the COVID-19 pandemic. (**a**) rescheduled doctor visits, (**b**) reasons for reduced pregnancy visits.

Rescheduled pregnancy visits were experienced as follows: three of them many times (2%); 31 people faced this one to two times (21%), and 113 of the respondents (77%) did not face this (Figure 1a).

To the question about affecting the frequency of pregnancy monitoring consultations, the respondents of the study were divided into four categories associated with the answers: 130 of them (88%) did not encounter difficulties with the frequency of consultations following the specialist’s recommendations; nine (6%) of the respondents had limited surveillance due to restrictions against the spread of the virus; four (3%) people encountered difficulties due to personal reluctance not to come into contact with the virus; while four (3%) of them faced the doctor’s reluctance not to come into contact with the virus (Figure 1b).

Regarding the TORCH panel: 119 of the study participants (84%) were tested; eight people from the study sample (2%) only partially, and 20 respondents (14%) did not perform the panel tests. Sixteen respondents replied that the reason for not performing the complete panel differed from the preventive measures against COVID-19.

Regarding infection with the SARS-CoV-2 virus, 120 of the respondents were not infected (82%) during pregnancy, 22 were infected, showing symptoms (15%), and five were asymptomatic (3%). Of the symptomatic ones, two had severe, twelve had moderate, and seventeen had mild symptoms.

Most participants followed the preventive measures (87%), while 12% only followed them partially, and the rest did not.

Depending on the health facility where the birth took place, the respondents of the study are divided as follows: 66 of them chose to give birth in a state health facility (45%); 79 people in a private health facility (54%); and two of the respondents chose the option “other places”.

Eleven participants felt influenced by the imposed measures towards preferring cesarean delivery.

In the case of natural birth, wearing a mask during labor and delivery, the discomfort and anxiety caused by wearing a mask were quantified as follows: 11 people felt a considerable impact due to wearing a mask during delivery (33%); two people were strongly affected by wearing the mask (6%); eight people felt discomfort or moderate anxiety (23%); two people felt slight discomfort or anxiety (6%); five people did not feel any discomfort or anxiety (15%), and five people (15%) did not wear a mask during the birth act.

**Figure 2:**
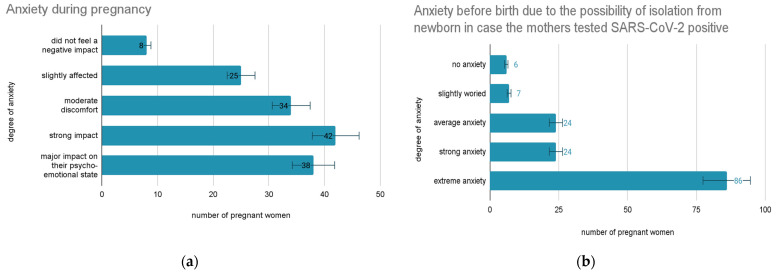
Anxiety reported by pregnant women (**a**) during pregnancy and (**b**) before birth because of the possibility of isolation from the newborn in case they tested positive.

Regarding the anxiety caused by the pandemic context, the respondents felt a negative impact on their own psychological and emotional state during pregnancy as follows: eight people did not feel a negative effect (5%); 25 people were slightly affected (17%); 34 people felt moderate discomfort (23%); 42 people felt a strong impact (29%), and 38 people perceived a significant effect on their psycho-emotional state (26%) (Figure 2a).

The degree of anxiety before birth because of the possibility of isolation from the newborn can be quantified as follows: six people did not feel anxiety related to possible isolation from the child (4%); seven people were slightly worried or anxious (5%); 24 of the mothers quantified anxiety as average (16%); 24 of the mothers considered the anxiety produced by the possible isolation of the child strong (16%) and 86 of the people, the majority, felt anxiety or worry with a considerable impact (59%) (Figure 2b).

We ran several statistical tests with anxiety produced by pandemic context and the fear of being infected with the pandemic virus as the dependent variables for consecutive analysis, trying to find the factors that anxiety may influence. We considered one as the lowest level of anxiety and five as the highest level. Fisher’s exact test showed no correlation between the level of anxiety generated by the pandemic and actual infection with SARS-CoV-2. When analyzing according to the exact level of anxiety, there was also no correlation, see Table 2.

For categorial data, as we had in our research, the Odds Ratio was calculated. We obtained OR = 0.83, which indicates 0.83 more chances that the pregnant women with an anxiety level of 4–5 would be SARS-CoV-2 infected compared to the pregnant women with a 1–3 anxiety level.

When analyzing anxiety versus vaccination and TORCH testing there was also no correlation; anxiety produced by the pandemic did not influence the level of vaccination, *p* = 0.4480, or the degree of TORCH testing, *p* = 0.4536, in the studied pregnant women.

However, when we calculated the Odds Ratio, we obtained OR = 1.92, so there are 1.92 more chances that the pregnant women with a higher level of anxiety such as 4–5 would get vaccinated compared to the pregnant women with an anxiety level of 1–3. For TORCH testing, the OR = 1.14, so there are 1.14 more chances that the pregnant women with higher levels of anxiety, 4–5, would skip TORCH testing than the pregnant women with 1–3 anxiety levels.

We also evaluated the decision to have a C-section versus natural birth according to the degree of anxiety in general; *p* = 0.4793 and we found no correlation.

The obtained OR was 1.15, so there are 1.15 more chances that the women with an anxiety level of 4–5 would give birth by C-section compared to women with lower levels of anxiety, such as 1–3.

In the case of positivity, isolation from the child for two weeks was implemented as a preventive measure against the spread of the SARS-CoV-2 virus. All mothers quantified the anxiety produced during the isolation period as having a considerable impact, representing all six mothers who tested positive for hospitalization. One of the mothers faced reduced lactation, breastfeeding being difficult; two of the mothers experienced reduced lactation but no impairment of breastfeeding.

Fifty-one people from the study sample considered that the absence of family had a considerable impact on emotional health (35%); 38 of the respondents thought that the lack of loved ones had a substantial impact on their psycho-emotional state (26%); 26 of the mothers considered the impact of this preventive measure to be medium (18%); 15 of the respondents thought that the impact of the lack of family was weak (11%); and 14 did not feel any impact on psycho-emotional health following this preventive measure (10%).

**Figure 3:**
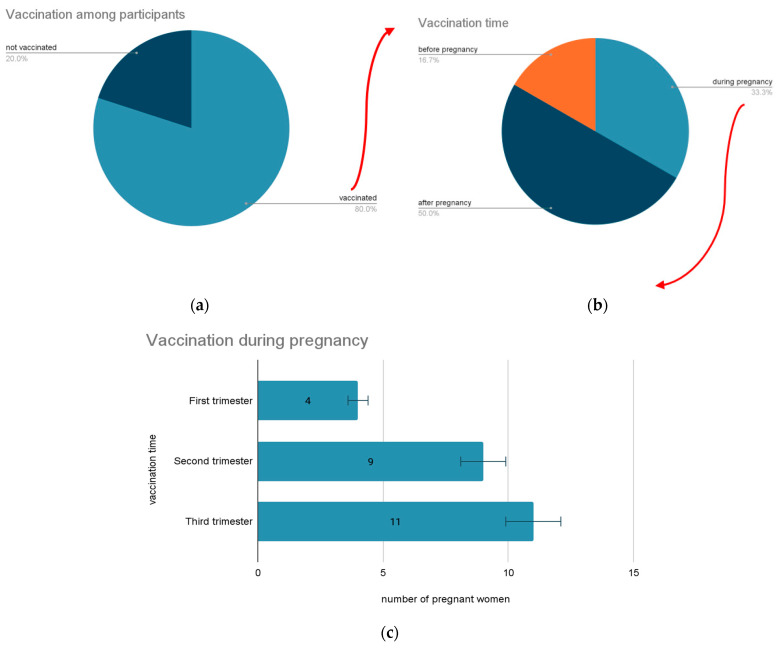
Vaccination uptake among participants and moment of immunization. (**a**) Number of vaccinated participants, (**b**) vaccination time, (**c**) the number of vaccinated participants depending on their pregnancy trimester for women vaccinated during pregnancy.

From the point of view of vaccination against COVID-19, the respondents were divided into 80% vaccinated (118 women) and 20% unvaccinated (29 women) (Figure 3a).

From the perspective of choosing the right moment for vaccination against COVID-19 depending on pregnancy, most of the respondents, 81 (55%), decided to be vaccinated after pregnancy. Thirteen (9%) women chose to be vaccinated before pregnancy, and 24 (16%) during pregnancy (Figure 3b). Of the women who were vaccinated during pregnancy, four (3% of the entire sample) did so in the first trimester, nine (6% of the whole sample) in the second trimester, and eleven (7% of the whole sample) in the third (Figure 3c).

When asked about motivations for vaccination against COVID-19, it emerged that for 105 of the 118 women who got vaccinated, the primary motivation was personal protection, followed immediately by the safety of the baby, with 88 women out of 105 selecting this option. The following reasons mattered to far fewer female respondents: 44 selected doctors’ recommendations as a motivation; 23 for visiting family under the officially recommended conditions; 20 for access to public spaces; and 13 for the ability to go on vacation. Weaker motivations were pressure from employers for three women, the ability to travel for business for two; protecting people in general, organizing a baptism, media recommendation, and government recommendation represented motivations for only one person each.

Following vaccination, 63 respondents who chose this prevention method reported minor local symptoms such as inflammation and pain at the injection site. Thirty-one of these did not experience any adverse effects following vaccination. Twenty-nine women reported generalized but short-term symptoms, while one reported long-term generalized symptoms. None of the vaccinated respondents reported an adverse effect on the fetus.

Among the 29 respondents who did not choose to be vaccinated against COVID-19, the two main reasons for avoiding vaccination, each for 22 of the respondents, was the lack of vaccine studies on pregnant women and the fear of adverse effects on the fetus.

Sixty-two participants noted an increase in their use of social media platforms, 31 reported an increase in overall caloric intake, with 25 noting an increase specifically in foods high in sugar, salt, and/or fat. Fourteen women noted an increase in tobacco smoking and nine in the consumption of alcoholic beverages. Six people observed excessive use of electronic devices.

Among the respondents, 36 (24%) reported a minor increase in the frequency of conflicts, 25 (17%) a noticeable increase, and nine (6%) reported a significant increase in the intensity and/or frequency of conflicts with their partner or relatives.

## 4. Discussion

Anxiety in pregnancy can be increased by various factors, from common ones—pregnancy related—to more general ones which might influence the entire population—war, pandemic or pollution [5]. Various studies have evaluated the anxiety caused by COVID-19 in pregnancy in different countries [5]. This supports the importance of our study to international literature, offering the opportunity to have a more global view, by including the results from our country as well [5].

Our results are consistent with other studies conducted during the COVID-19 pandemic that demonstrate high levels of anxiety in expectant women from various countries and geographic, cultural, or sociopolitical backgrounds, such as Canada, the United States, India, Israel, the United Kingdom, Australia, and China [18,19,20].

### 4.1. SARS-CoV-2 Symptoms in Pregnancy

COVID-19 in pregnancy is a new condition, and it is important to study all possible factors that influence the outcome of the pregnancy [21]. In some studies, women with COVID-19 during pregnancy frequently appeared with fever and coughing [11]. Most COVID-19 symptoms in pregnant women were mild to moderate, and most appeared during the third trimester of pregnancy [13].

Pregnancy is a stressful period due to various changes and even before the COVID-19 pandemic, studies have shown the need for special care for this category by testing and monitoring during the pregnancy period as well as before becoming pregnant [22].

### 4.2. Anxiety of Pregnant Women in COVID-19 Pandemic

The COVID-19 pandemic may raise the risk of mental health issues, especially in susceptible populations, such as pregnant women, who must take extra measures [23]. These factors include travel limitations, isolation, social isolation, limited access to medical care, and chronic daily life changes [23]. It can be difficult just to be pregnant. Pregnant women frequently face stress due to their impending transition to parenthood, worries about their health and the health of their unborn child, and uncertainty about the future even in the absence of a pandemic [23]. The pandemic’s novelty and unpredictability may increase worries and make it more difficult for pregnant women’s psychological wellbeing [23].

Important maternal–fetal repercussions result from prenatal anxiety [10]. Excessive panic would probably worsen the symptoms, leading in some cases to consequences such as coronasomnia [4,15]. This may be linked to preterm birth, fetal growth restriction, and obstetric difficulties and may have long-lasting impacts on a child’s growth and development, either directly or indirectly [10]. Deliveries, postpartum, breastfeeding, and solitude brought on by restrictive measures taken against COVID-19 were also linked to higher levels of anxiety [24]. Contrary to what we expected from some Spanish studies, being a single parent reduced the level of anxiety towards COVID-19 [24]. Children who experience maternal anxiety during pregnancy are more likely to develop various neuropsychiatric disorders, such as attention deficit/hyperactivity disorder [10].

The degree of anxiety can be influenced by a person’s personality, resiliency, education, family support, satisfaction with the quality of their lives, occupation, and financial situation [25]. Anxiety may be impacted by people’s knowledge, attitudes, and practices regarding COVID-19 during this pandemic [25]. In cities and nations with significant outbreaks, for instance, the general population and a subpopulation have reported higher anxiety levels [25].

Although anxiety may take many forms, such as eco-anxiety [26], or war related anxiety [27], one form of anxiety was generated by the COVID-19 pandemic [28].

Due primarily to the effects associated with the climate change problem, some governments, such as Italy, have concentrated on establishing national climate change adaptation plans over the past ten years [1]. Younger people are generally more concerned about this matter and this includes pregnant people [1]. Regarding climate change, some persons may exhibit severe stress and related psychological symptomatology [1]. Eco-anxiety was linked to anxiety and depressive symptoms, self-efficacy, awareness of environmental issues, and pro-environmental behaviors, according to an Italian study [1].

Other causes of anxiety may include war situations as well, the most recent one being the Ukrainian–Russian war [1]. Some studies have evaluated this cause of anxiety as well, and in our country this emergency may have caused anxiety in the general population, especially in pregnant women since we are at the border [1]. This aspect was not evaluated in our study since we had finished our survey data collection at the moment when the war was starting, and anxiety with other causes was not the main objective of our study, only the SARS-CoV-2 pandemic [1].

In Romania, in the case of mothers who tested positive for the SARS-CoV-2 virus, as a preventive measure, the isolation of the child from the mother for two weeks was implemented. Testing for COVID-19 in pregnancy represents a tool for prevention [21]. This fact can cause restlessness or anxiety for the mother. Regarding the bond between mother and child, the impact of their isolation for two weeks was quantified by the SARS-CoV-2-positive mothers as having a considerable impact on the formation of this bond. The fact that many women are given false information about the safety of COVID-19 and breastfeeding, which results in infants being taken away from their mothers or mothers being forbidden from nursing when they develop COVID symptoms after giving birth, is a major concern [29]. In the early phases of the epidemic, mothers and newborns were routinely separated in a number of nations, and breast milk replacements were occasionally advised [29]. Similar advice was given by the American Academy of Pediatrics, which has since been modified, to separate the mother and child if a case was suspected (AAP, 2020) [29]. Even though numerous UK organizations have stated that breastfeeding is safe and should be supported (UNICEF UK, 2020), concerned parents and medical professionals may have been misled by these false claims [29].

Unfortunately, studies performed on this subject highlighted that this measure had a negative impact and in most of the cases it did not prevent the infection [30]. Some studies estimated that 125,000 lives could have been saved if mothers and babies were not separated [30].

In our study, one of the unfortunate consequences of the stress generated by the pandemic and the measures to combat it, especially the lockdowns, has been increased tension between family members and domestic conflicts. As a result of the stress during the pandemic, there has been an increase in the frequency of certain behaviors generally associated with stress reduction or used as a coping mechanism, such as the consumption of alcohol or recreational drugs, among the population.

Research conducted in Wuhan showed that participants’ second-trimester anxiety prevalence was highest (21.1%), third-trimester anxiety prevalence was lowest (20.7%), and first-trimester anxiety prevalence was intermediate (20.9%) [25].

In our group of participants, the anxiety might have been increased by the decision to restrict maternity hospital visits. The lack of loved ones can affect mental and emotional health. For this reason, the ban’s impact on visitors’ access to the hospital, as a preventive measure against the SARS-CoV-2 virus, might have played an important role in the increased anxiety. Infants’ initial and only natural source of appropriate nutrition, particularly during the first six months of life, is breastmilk [31]. The World Health Organization advises mothers with either suspected or confirmed COVID-19 to commence or continue nursing, stressing that the advantages of doing so far exceed the potential risks of transmission [31].

### 4.3. Monitorization of Pregnancy in COVID-19 Pandemic

Monitoring the pregnancy is also a priority for the proper development of the act of birth and the newborn’s well-being [32]. In the context of the pandemic, pregnancy monitoring could be disrupted by the reorganization of the medical system [32]. To determine the condition of the fetus and, implicitly, of the mother, it is important to carry out the tests related to the TORCH panel to detect possible infections early [32].

Most women in a study conducted in Wuhan closely followed the official COVID-19 news and were unconcerned about becoming sick from the echography transducer [25]. As suggested by the government, over two-thirds of the participants either delayed or decreased the frequency of their prenatal checkups [25]. However, a high percentage of pregnant women—10.2%—were anxious about picking up COVID-19 through the echography transducer [25]. In our group of participants, a few women had reduced the frequency of checkups because of the COVID-19 restrictions or reluctance from them or the doctor. It is concerning that 20 participants did not perform the TORCH panel, and more alarming is that, for 16 of them, the reason was not COVID-19, a problem encountered in our country even before the pandemic.

Studies conducted before the pandemic showed significant differences, *p* < 0.001, in monitoring pregnant women according to age group, so, a Romanian study showed in a group of adolescent childbearing women a mean of 3.52 ± 4.56 controls performed during all pregnancies, compared to 7.34 ± 5.75 controls in an older control group [33].

Monitoring of pregnant women before and after the COVID-19 pandemic must be regarded also from the comorbidities point of view, as there are conditions that may influence the outcome of pregnancy other than infection with SARS-CoV-2 [34].

### 4.4. Preventive Measures Taken—Outcomes and Influences upon Pregnancy

In Romania, preventive measures, such as isolation from the newborn in the case of positive SARS-CoV2 testing in mothers, might have had a negative impact on the mother, more precisely affecting lactation, through the lack of stimulation from the newborn.

When an infant is taken away from its mother, it may be more likely that the youngster will become infected with other dangerous infections [31]. During the COVID-19 pandemic, many lactating mothers may stop or reduce the time they exclusively breastfeed their babies, which could have more serious repercussions [31]. These factors include fear of the risk of transmission, a lack of knowledge, an unfavorable attitude, or inappropriate practice [31]. During COVID-19, in an Ethiopian study, 487 mothers (78.4%) reported feeling positive about solely breastfeeding their infants. In the same way, three-quarters (74.9%) of mothers reported that it felt pleasant to breastfeed their children whenever they needed to during the epidemic [31]. Additionally, 395 (63.6%) mothers reported feeling at ease using COVID-19 safeguards while nursing their infants [31].

Breastfeeding mothers had struggled to find help during the epidemic and have encountered several difficulties that forced them to stop nursing before they were ready [10]. During the COVID-19 epidemic, several individual and environmental factors interacted to affect breastfeeding outcomes [10]. Lockdowns and the isolation, stress, and grief they cause have a detrimental psychological effect on nursing mothers [10]. Further evidence that worries may be connected to early weaning is that non-breastfeeding women scored more fearfully on the COVID-19 Scale (FCV-19S) [10]. Decisions about infant feeding may also be significantly influenced by the acceptability of vaccinations and a thorough understanding of their safety [10].

Mothers may not have received the necessary help because in-person lactation support was scarce in the context of COVID-related mitigation initiatives and policies [35]. If left unattended, this lack of, or weakened, assistance may harm the start and continuation of breastfeeding [35]. Due to the difficulty of interacting with people in person and the lack of access to daycare, the pandemic has severely influenced mothers’ capacity to access breastfeeding support [35]. Due to a lack of support, first-time mothers may be more susceptible to early breastfeeding termination [35]. However, giving mothers extra time at home with their children has also positively impacted nursing [35]. During COVID-19, resources are required to assist with breastfeeding in the workplace [35].

Higher-educated women do not always have better knowledge, attitudes, or behaviors regarding effective COVID-19 prevention and control methods in the Democratic Republic of Congo (DRC) [36]. The findings suggest that education can influence COVID-19 burden reduction in both good and negative ways [36]. As a result, women’s education level does not significantly affect a range of knowledge, attitudes, and practice variables in the DRC, including evaluating efficacy and the risk of contracting COVID-19 [36]. This is similar to our findings, where most women had university degrees and were vaccinated against COVID-19 (80%) but still presented great anxiety levels due to all the other preventive measures taken during the pandemic. At the same time, with influencing their anxiety level, it may have helped them to better follow the prevention recommendations.

For instance, women with at least a secondary education are far less likely to receive a COVID-19 vaccination. They are ready to keep it a secret if a family member contracts COVID-19 because they think the vaccine is safer and more effective [36].

Although most women in an Iranian study understood COVID-19, there are disparities in knowledge based on factors including age, education, place of residence, and other factors [37]. Mothers had higher knowledge if they knew someone who had died or recovered from COVID-19 [37]. Additionally, they used appropriate disease-preventive techniques [37]. Even though the practice was generally good, most of them were concerned about the long pandemic phase and had some information misconceptions [37].

In an Iranian study, the majority of study participants—roughly 3/4—had solid awareness of the COVID-19 epidemic [38]. However, over 2/5 of the participants had a negative outlook and exhibited inadequate COVID-19 prevention practices [38]. This would put everyone at high risk of contracting COVID-19, including mothers, other family members, and the general public [38]. Regarding the mothers undergoing natural birth from our study and implicitly wearing the mask throughout the act of birth, most of the study’s respondents felt that the whole process was more difficult.

As a result of the coronavirus pandemic, this would significantly deteriorate the profile of maternal morbidity and mortality [38]. Health education platforms geared towards enacting and cultivating COVID-19-related knowledge, attitudes, and practices are now necessary, especially for mothers with limited access to information because of their domestic responsibilities [37]. In an Ethiopian study, 52.29% of pregnant women practiced COVID-19 infection prevention [39]. The results are lower than those of studies conducted in South Africa (76%), India (92.7%), and China (89.7%) [39]. Pregnant women with secondary or higher educational status had a 3.36 times greater likelihood of using effective COVID-19 infection prevention strategies than those with no formal education [39].

It is advised that every immunization program must be trustworthy to be effective [40]. Additionally, it is equally important that the officials ensure vaccination dosages are secure, have judicious vaccine administration and effective management, and convince the general public of vaccines’ safety and effectiveness while inspiring them to accept them [7,40]. Maternal IgG can pass through the placenta and give the newborn immunity [16]. In a study on immune response after vaccinating women against SARS-CoV2, pregnant, nursing, and non-pregnant controls all had robust and comparable IgG titers that were all noticeably greater than those seen in pregnant women with prior SARS-CoV-2 infection [16]. Boosting led to increased blood levels of IgG, which were then transferred to the baby through the placenta and breast milk [16].

The main intermediary between pre-existing information and actual COVID-19 immunization practice can be attitude [40]. These results are the foundation of public health awareness campaigns that must work to change people’s attitudes in a favorable direction [40].

To slow the spread of SARS-CoV-2 and prevent the creation of new strains, one of the most effective and affordable public health intervention techniques is the COVID-19 vaccine [15]. Although it is infrequent, vertical transmission has also been seen in a few cases in pregnant women with SARS-CoV-2 [15]. It is difficult to confirm transmission early in the pregnancy without a phenotype, as with congenital Zika or rubella syndrome, or increased miscarriages, as there is little information available on intrauterine infection earlier in pregnancy with the resolution of maternal infection before the time of delivery [37]. SARS-CoV-2 is also not known to cause chronic infection. Miscarriage due to SARS-CoV-2 infection can happen at 34 weeks [37]. Therefore, it has been discovered that immunization against COVID-19 is the most effective method for preventing major disease or consequences in pregnant women (and the fetus) [4].

A factor that could have influenced this decision was the availability of vaccination, which was not offered to the general population in Romania until 2021 [7]. We support COVID-19 vaccination during pregnancy. The emergency design and testing of vaccines was a reason for avoiding them for 19 women, while the fear of side effects was important for 17 of them. In other studies, medical and social workers in Romania still have reservations about the SARS-CoV2 vaccine; as a result, these reservations may be shared by the general populace, who could benefit from focused, targeted public health campaigns [8]. Less significant reasons were the perception of vaccine effectiveness as insufficient for nine women, mistrust of the government for nine women, mistrust of the media, which promoted the vaccine, for eight women, and mistrust of pharmaceutical companies for eight women. Finally, three of the respondents did not consider themselves at risk for COVID-19.

### 4.5. Limitation of the Study

The survey has several limitations. Initially, our study is limited by its cross-sectional design and the inclusion and exclusion criteria we applied. We included women older than 18 years who gave birth or were pregnant in the period 2020–2022 when the SARS-CoV-2 virus occurrence forced authorities to take different sanitary measures. We excluded women who were unable to read and understand the questionnaire and women with severe physical conditions (severe hypertension, diabetes mellitus, cardiac disease, kidney or liver disease), severe pregnancy-related conditions (for example, antepartum hemorrhage, pre-eclampsia, hyperemesis gravidarum, premature rupture of membranes), and/or severe mental conditions. Another limitation is the small number of the studied group, 147 participants, and the majority had university degrees. The high educational degree might have improved the use of preventive measures and may have made them more prone to go to the medical check-ups and recommended tests. In our country, the lockdown measures varied during the time when the study was performed; considering the multiple and rapid changes in legislation and recommendations and the small number of included participants in this study, an analysis based on the lockdown recommendations was not performed.

In Romania, the waves of pandemic occurred with a delay of several months compared with the European Union and the exact distribution of the strains in the general population was not properly documented, thus making it impossible to correlate the outcome of our study with the prevalence of SARS-CoV-2 strains [7,8,41].

### 4.6. Future Developments

COVID-19 was a unique pandemic but the changing times we are living in have shown that the unexpected may happen. Future developments need to be predicted in order for governments and people to be prepared. For better preparedness, we suggest that the pregnant women category should be considered separately in projects and plans.

## 5. Conclusions

Although, in general, many medical services were diminished, all pregnancies in the studied group were monitored. The level of education of the studied pregnant women influenced the attendance to the gynecologist. Gynecologists followed almost all the pregnancies; approximately a quarter of them had rescheduled pregnancy visits, and the important panels were not omitted.

Most pregnant women felt anxiety caused by the pandemic, and the level of anxiety influenced their vaccination decisions, the TORCH testing, and the method of giving birth The women followed SARS-CoV2 preventive recommendations. Maternal psychological health problems and anxiety may have important impacts on the child’s health and its development; thus, greater consideration should be offered to this vulnerable group in special situations such as war, conflicts, climatic changes and pandemics.

Fortunately, only a small proportion of our study participants were infected with the coronavirus, and there were no consequences noticed for the fetuses at birth.

Healthcare systems should consider the results and conclusions of similar studies when designing procedures and protocols to manage future pandemics. With good informative programs and carefully implemented preventive measures, even in stressful situations and periods of anxiety, such as during a pandemic, pregnant women can be protected. Healthcare providers can reduce anxiety levels and suppress all the unfavorable outcomes by offering information and empathy. Explaining the reality is the key to success and understanding.

## Figures and Tables

**Table 1:** General, socio-demographic, educational, and pregnancy status of respondents to the questionnaire.

Characteristics	N = 147(%)
Family status (married)	127 (86.4%)
Domain of activity (employed)	140 (95.2%)
Geographic area (urban)	140 (95.2%)
Post-secondary school studies	4 (2.7%)
University / Post-university studies	143 (97.3%)
Pregnancy (monitored)	147 (100%)
Birth (C-section)	33 (22.45%)
SARS-CoV-2 infected during pregnancy	27 (18.36%)
Isolated at birth 2 weeks	6 (4.08%)
Vaccinated against COVID-19	118 (80%)
Anxiety in the pandemic context	141 (95.91%)

**Table 2:** Correlation of the level of anxiety and SARS-CoV-2 infection in the studied group, *p* values.

Level of Anxiety	1–2	2–3	3–4	4–5
SARS-CoV-2 infected/not infected	0.7575	0.4314	0.3764	0.3982

## Data Availability

Not applicable.

## Supplementary Materials

The following supporting information can be downloaded at: https://www.mdpi.com/article/10.3390/vaccines11010035/s1, File S1: STROBE checklist; Supplementary Materials File S2: Questionnaire and informed consent.

## References

[1] Barchielli B., Cricenti C., Gallè F., Sabella E.A., Liguori F., Da Molin G., Liguori G., Orsi G.B., Giannini A.M., Ferracuti S. (2022). Climate Changes, Natural Resources Depletion, COVID-19 Pandemic, and Russian-Ukrainian War: What Is the Impact on Habits Change and Mental Health?. Int. J. Environ. Res. Public. Health.

[2] Gheorghe A.S., Negru Ş.M., Nițipir C., Mazilu L., Marinca M., Gafton B., Ciuleanu T.E., Schenker M., Dragomir R.D., Gheorghe A.D. (2021). Knowledge, Attitudes and Practices Related to the COVID-19 Outbreak among Romanian Adults with Cancer: A Cross-Sectional National Survey. ESMO Open.

[3] Cocoş R., Mahler B., Turcu-Stiolica A., Stoichiță A., Ghinet A., Shelby E.-S., Bohîlțea L.C. (2022). Risk of Death in Comorbidity Subgroups of Hospitalized COVID-19 Patients Inferred by Routine Laboratory Markers of Systemic Inflammation on Admission: A Retrospective Study. Viruses.

[4] Oțelea M.R., Zugravu C., Rașcu A., Arghir O.C., Manolescu L.S.C., Mates D. (2022). Coronasomnia in Employees without a Direct Contact with COVID-19 Infected Patients in Their Workplace. Healthcare.

[5] Morris J.R., Jaswa E., Kaing A., Hariton E., Andrusier M., Aliaga K., Davis M., Cedars M.I., Huddleston H.G. (2022). Early Pregnancy Anxiety during the COVID-19 Pandemic: Preliminary Findings from the UCSF ASPIRE Study. BMC Pregnancy Childbirth.

[6] Maternal Mental Health. https://www.who.int/teams/mental-health-and-substance-use/promotion-prevention/maternal-mental-health.

[7] Manolescu L.S.C., Zaharia C.N., Dumitrescu A.I., Prasacu I., Radu M.C., Boeru A.C., Boidache L., Nita I., Necsulescu A., Medar C. (2022). COVID-19 Parental Vaccine Hesitancy in Romania: Nationwide Cross-Sectional Study. Vaccines.

[8] Manolescu L.S.C., Zaharia C.N., Dumitrescu A.I., Prasacu I., Radu M.C., Boeru A.C., Boidache L., Nita I., Necsulescu A., Chivu R.D. (2021). Early COVID-19 Vaccination of Romanian Medical and Social Personnel. Vaccines.

[9] Serra F.E., Rosa Junior E.R., de Rossi P., Francisco R.P.V., Rodrigues A.S. (2022). COVID-19: Impact of Original, Gamma, Delta, and Omicron Variants of SARS-CoV-2 in Vaccinated and Unvaccinated Pregnant and Postpartum Women. Vaccines.

[10] Staneva A., Bogossian F., Pritchard M., Wittkowski A. (2015). The Effects of Maternal Depression, Anxiety, and Perceived Stress during Pregnancy on Preterm Birth: A Systematic Review. Women Birth J. Aust. Coll. Midwives.

[11] Mahler B., Croitoru A. (2019). Pulmonary rehabilitation and tuberculosis: A new approach for an old disease. Pneumologia.

[12] Zaigham M., Andersson O. (2020). Maternal and Perinatal Outcomes with COVID-19: A Systematic Review of 108 Pregnancies. Acta Obstet. Gynecol. Scand..

[13] Wang C., Zhou Y.-H., Yang H.-X., Poon L.C. (2020). Intrauterine Vertical Transmission of SARS-CoV-2: What We Know so Far. Ultrasound Obstet. Gynecol..

[14] Sharps M.C., Hayes D.J.L., Lee S., Zou Z., Brady C.A., Almoghrabi Y., Kerby A., Tamber K.K., Jones C.J., Adams Waldorf K.M. (2020). A Structured Review of Placental Morphology and Histopathological Lesions Associated with SARS-CoV-2 Infection. Placenta.

[15] Abebe E.C., Tiruneh G.A., Adela G.A., Ayele T.M., Muche Z.T., T/Mariam A.B., Mulu A.T., Zewde E.A., Baye N.D., Dejenie T.A. (2022). COVID-19 Vaccine Uptake and Associated Factors among Pregnant Women Attending Antenatal Care in Debre Tabor Public Health Institutions: A Cross-Sectional Study. Front. Public Health.

[16] Gray K.J., Bordt E.A., Atyeo C., Deriso E., Akinwunmi B., Young N., Baez A.M., Shook L.L., Cvrk D., James K. (2021). Coronavirus Disease 2019 Vaccine Response in Pregnant and Lactating Women: A Cohort Study. Am. J. Obstet. Gynecol..

[17] Checklists. https://www.strobe-statement.org/checklists/.

[18] Ali N.A., Feroz A.S. (2020). Maternal Mental Health Amidst the COVID-19 Pandemic. Asian J. Psychiatry.

[19] Taubman-Ben-Ari O., Chasson M., Sharkia S.A., Weiss E. (2020). Distress and Anxiety Associated with COVID-19 among Jewish and Arab Pregnant Women in Israel. J. Reprod. Infant Psychol..

[20] Davenport M.H., Meyer S., Meah V.L., Strynadka M.C., Khurana R. (2020). Moms Are Not OK: COVID-19 and Maternal Mental Health. Front. Glob. Womens Health.

[21] Alzamora M.C., Paredes T., Caceres D., Webb C.M., Valdez L.M., La Rosa M. (2020). Severe COVID-19 during Pregnancy and Possible Vertical Transmission. Am. J. Perinatol..

[22] Orzechowski M., Timmermann C., Woniak K., Kosenko O., Mikirtichan G.L., Lichtshangof A.Z., Steger F. (2021). Access to Prenatal Testing and Ethically Informed Counselling in Germany, Poland and Russia. J. Pers. Med..

[23] Ilska M., Brandt-Salmeri A., Kołodziej-Zaleska A., Preis H., Rehbein E., Lobel M. (2022). Anxiety among Pregnant Women during the First Wave of the COVID-19 Pandemic in Poland. Sci. Rep..

[24] Esteban-Gonzalo S., Caballero-Galilea M., González-Pascual J.L., Álvaro-Navidad M., Esteban-Gonzalo L. (2021). Anxiety and Worries among Pregnant Women during the COVID-19 Pandemic: A Multilevel Analysis. Int. J. Environ. Res. Public Health.

[25] Ding W., Lu J., Zhou Y., Wei W., Zhou Z., Chen M. (2021). Knowledge, Attitudes, Practices, and Influencing Factors of Anxiety among Pregnant Women in Wuhan during the Outbreak of COVID-19: A Cross-Sectional Study. BMC Pregnancy Childbirth.

[26] Pihkala P. (2018). Eco-Anxiety, Tragedy, and Hope: Psychological and Spiritual Dimensions of Climate Change. Zygon®.

[27] Cricenti C., Mari E., Barchielli B., Quaglieri A., Burrai J., Pizzo A., D’Alessio I., Giannini A.M., Ferracuti S., Lausi G. (2022). Can Emotion Regulation Affect Aggressive Responses? A Study on the Ukrainian-Russian Conflict in a Non-Directly Exposed Sample. Int. J. Environ. Res. Public Health.

[28] Giudice V., Iannaccone T., Faiella F., Ferrara F., Aversano G., Coppola S., De Chiara E., Romano M.G., Conti V., Filippelli A. (2022). Gender Differences in the Impact of COVID-19 Pandemic on Mental Health of Italian Academic Workers. J. Pers. Med..

[29] Study of Anxiety, Fear and Depression Associated with Breastfeeding in COVID-Positive Mothers. https://www.jsafog.com/abstractArticleContentBrowse/JSAFOG/25952/JPJ/fullText.

[30] New Research Highlights Risks of Separating Newborns from Mothers during COVID-19 Pandemic. https://www.who.int/news/item/16-03-2021-new-research-highlights-risks-of-separating-newborns-from-mothers-during-covid-19-pandemic.

[31] Gebretsadik G.G., Tadesse Z., Mamo L., Adhanu A.K., Mulugeta A. (2022). Knowledge, Attitude, and Determinants of Exclusive Breastfeeding during COVID-19 Pandemic among Lactating Mothers in Mekelle, Tigrai: A Cross Sectional Study. BMC Pregnancy Childbirth.

[32] Radu M.C., Boeru A.C., Marin M., Manolescu L.S. Diabetes Mellitus and Pregnancy. Proceedings of the International Conference on Interdisciplinary Management of Diabetes Mellitus and its Complications INTERDIAB 2020.

[33] Radu M.C., Manolescu L.S., Chivu R., Zaharia C., Boeru C., Pop-Tudose M.-E., Necsulescu A., Otelea M. (2022). Pregnancy in Teenage Romanian Mothers. Cureus.

[34] Niță I., Nițipir C., Toma S.A., Limbău A.M., Pîrvu E., Bădărău I.A., Suciu I., Suciu G., Manolescu L.S.C. (2021). Correlation between Androgen Receptor Expression and Immunohistochemistry Type as Prognostic Factors in a Cohort of Breast Cancer Patients: Result from a Single-Center, Cross Sectional Study. Healthcare.

[35] Snyder K., Worlton G. (2021). Social Support During COVID-19: Perspectives of Breastfeeding Mothers. Breastfeed. Med..

[36] Loleka B.Y., Ogawa K. (2022). Influence of the Level of Education on Women’s Knowledge, Attitude, and Practices to Control the Transmission of COVID-19 in the Democratic Republic of the Congo. Sci. Afr..

[37] Shahbaznejad L., Navaeifar M.R., Movahedi F.S., Hosseinzadeh F., Fahimzad S.A., Shirazi Z.S., Rezai M.S. (2021). Knowledge, Attitude and Practice of Sari Birth Cohort Members during Early Weeks of COVID-19 Outbreak in Iran. BMC Public Health.

[38] Goshiye D., Abegaz Z., Gedamu S. (2022). Knowledge, Attitude, and Practice towards COVID-19 among Mothers in Dessie Town, Northeast Ethiopia, 2020. Interdiscip. Perspect. Infect. Dis..

[39] Mose A., Zewdie A., Sahle T. (2022). Pregnant Women’s Knowledge, Attitude, and Practice towards COVID-19 Infection Prevention in Ethiopia: A Systematic Review and Meta-Analysis. PLoS ONE.

[40] Sengupta M., Dutta S., Roy A., Chakrabarti S., Mukhopadhyay I. (2022). Knowledge, Attitude and Practice Survey towards COVID-19 Vaccination: A Mediation Analysis. Int. J. Health Plann. Manage..

[41] Radu M.C., Boeru C., Marin M., Manolescu L.S. (2021). SARS-CoV-2 Infection in Seven Childbearing Women at the Moment of Delivery, a Romanian Experience. Cureus.

