# Supplementary material for: SARS-CoV-2 Infection Anxiety, Knowledge and Attitudes in University Degree Pregnant Women from Romania—A Cross-Sectional Observational Survey in the First Two Pandemic Years
Source: Vaccines (Basel). 2022 Dec 23;11(1):35. doi: 10.3390/vaccines11010035 (PMC9864279; doi:10.3390/vaccines11010035)
Supplement: Supplementary file 1 [file vaccines-11-00035-s001.zip › Supplementary File S2 - Questionnaire and informed Concent.pdf]

INFORMED CONSENT/QUESTIONNAIRE ADDRESSED TO PERSONS OVER 18 YEARS OLD

We invite you to contribute to our research on Pregnant women’s knowledge, attitude, and practice towards COVID-19 infection, vaccination, prevention measures and the influence of pandemic over pregnancy monitoring. The questionnaire to be completed has several sections and is addressed to pregnant women over 18 years.

The time required to complete this questionnaire is approximately 15 minutes. Participation is voluntary and your answers will be treated confidentially, the information you provide will only be used for research purposes. You have the freedom at any time to withdraw from the study, to interrupt or to resume the questionnaire.

The research complies with the international ethical recommendations regarding the absolute confidentiality of the data collected in the study as well as the anonymity and safety of the respondents' data. The requirements of EU Regulation 2016/79, for the protection of individuals regarding the processing of personal data and the free movement of such data, are respected. The requirements of Law no. 506/2004, regarding the processing of personal data and the protection of private life in the electronic communications sector are respected. It is important that during the study you answer the questions as honestly as possible to draw correct conclusions.

For any clarification related to this study, during or after its completion you can contact us by email at loredana.manolescu@umfcd.ro

Questionnaire

1) Initials of name and surname:

2) Age:

- < 18 years
- 18-35 years
- > 35 years

3) Occupation:

- Pupil/Student
- household
- employed

4) Marital status:

- Married
- Single
- Divorced

5) Living environment:

- Rural
- Urban

6) Level of studies:

- No studies
- Secondary school cycle (8 grades)
- Compulsory education cycle (10 grades)
- High school cycle (12 grades)
- Post-secondary school
- University/Post university studies

7) The child will be raised by:

- Two parents
- A parent
- Legal guardian

8) Was the pregnancy monitored?

- Yes
- Not

9) If so, by whom was the pregnancy monitored?

- gynecologist
- general practitioner
- midwife

10) During your pregnancy, were your scheduled pregnancy monitoring consultations postponed or canceled due to the reorganization of the medical system during the pandemic?

- Yes, multiple times
- Yes, once or twice
- Not at all

11) Pregnancy surveillance during the SARS COV-2 pandemic was carried out (select that apply):

- Depending on the specialist's recommendations, without difficulty
- Less often than the specialist's recommendations, due to restrictions against the spread of the SARS COV-2 virus
- Less often than specialist recommendations, with reluctance on your part to avoid encountering the SARS COV-2 virus

- o Less often than the specialist's recommendations, with reluctance on his part, to avoid encountering the SARS COV-2 virus

12) During pregnancy, your contact with the SARS COV-2 virus was of the type:

- o I was infected and had symptoms
- o I was infected, but without symptoms
- o I was not infected

13) In the case of symptomatic COVID infection, the symptoms were:

- o severity
- o Media
- o Easy
- o With long-term sequelae

14) During pregnancy, consider that:

- o We followed the rules to prevent the spread of the virus
- o I did not follow the rules of prevention
- o I partially followed the rules of prevention

1 5) In the case of compliance with the rules to prevent the transmission of the virus, did you feel a sense of security and peace as a result?

- o Very strong
- o Strong
- o Moderate
- o Weak
- o Not at all

16) During the pregnancy period, the TORCH panel examination was carried out:

- o Total
- o Partial
- o Not at all

16') If the tests in the TORCH panel were not fully realized, to what extent did the prevention rules during the pandemic influence this fact?

- o They had a great influence
- o They had a partial influence
- o They had little influence
- o Not at all

17) The birth was achieved:

- o Naturally
- o By cesarean section

18) Due to the necessity of testing for the SARS-CoV-2 virus for hospitalization and repeating the test in case of prolongation of hospitalization, did you feel influenced to choose cesarean delivery?

- Yes
- Not

19) The birth took place in a:

- State institution
- Private institution
- Other

20) The birth took place in:

- Capital
- Province

21) In the case of a natural birth, will wearing a mask during labor and delivery cause anxiety and/or discomfort?

- Very strong
- Strong
- Moderate
- Weak
- Not at all

22) In the case of a natural birth, did you feel that wearing a mask during labor and delivery would make it difficult to participate and proceed?

- Very strong
- Strong
- Moderate
- Weak
- Not at all

23) Did the anxiety produced by the pandemic context (fear of infection with the SARS-CoV-2 virus) during pregnancy have a negative impact on your own psychological and emotional state?

- Very strong
- Strong
- Moderate
- Weak
- Not at all

24) Before the birth, knowing the possibility of isolating you from the baby for 2 weeks, in case of testing positive for the SARS-CoV-2 virus, would it cause you anxiety and/or worry?

- Very strong

- Strong
- Moderate
- Weak
- Not at all

25) If you tested positive for the perinatal SARS-CoV-2 virus, following isolation from the child for two weeks, your degree of anxiety can be quantified:

- Very strong
- Strong
- Moderate
- Weak
- Not at all

26) If you tested positive for the perinatal SARS-CoV-2 virus, following isolation from your child for two weeks, how much do you think this isolation affected the formation of the emotional bond between you and the baby?

- Very strong
- Strong
- Moderate
- Weak
- Not at all

27) If you tested positive for the perinatal SARS-CoV-2 virus, following isolation from the child for two weeks, how will it affect lactation?

- Lactation stopped, breastfeeding became impossible
- Lactation decreased, caused difficulties in breastfeeding
- Lactation decreased but returned to acceptable levels with breastfeeding
- Lactation decreased without significantly affecting breastfeeding
- Lactation was not affected

28) If you tested positive for the perinatal SARS-CoV-2 virus, following isolation from the child for two weeks, how will it affect the initiation and course of breastfeeding?

- Breastfeeding could no longer be initiated
- Breastfeeding was initiated with major difficulties
- Breastfeeding was initiated with minor difficulties
- Breastfeeding was initiated without difficulty
- I was not interested in breastfeeding

29) If you tested positive for the perinatal SARS-CoV-2 virus, following isolation from the child for two weeks, how was your psychological state affected? Have you experienced (select that apply):

- Sadness
- Lack of motivation

- Irritability
- Guilt, hopelessness
- Fatigue
- Restlessness or agitation
- Difficulty concentrating or making decisions
- Sleep disturbance
- Pain, cramps or digestive disorders without a clear physical cause
- Difficulty forming an attachment to the child
- Doubts about one's ability to care for the child

30) Does the lack of contact with the family during the maternity/hospital stay, especially after the birth, affect you psychologically/emotionally in a negative way?

- Very strong
- Strong
- Moderate
- Weak
- Not at all

3 1) How much do you consider that the measures to prevent the transmission of the SARS-CoV-2 virus during the pandemic affected the natural evolution of your pregnancy?

- Very strong
- Strong
- Moderate
- Weak
- Not at all

32) How much do you consider that the restrictions related to traveling outside the workplace, the hospital, supermarkets during the SARS-CoV-2 pandemic affected you psychologically during pregnancy?

- Very strong
- Strong
- Moderate
- Weak
- Not at all

33) How much do you consider that the regulations related to wearing a mask during the pandemic affected you psychologically during pregnancy?

- Very strong
- Strong
- Moderate
- Weak
- Not at all

34) How much do you think that wearing a mask during the pandemic directly affected your health during pregnancy?

- Very strong
- Strong
- Moderate
- Weak
- Not at all

35) How much do you think it will psychologically and emotionally affect the approach to birth in the medical system during the pandemic?

- Very strong
- Strong
- Moderate
- Weak
- Not at all

3 6) Have you been vaccinated against SARS-CoV-2?

- Yes
- Not

37) If you were vaccinated, in which trimester of pregnancy did you do it?

- Quarter I
- Quarter II
- Quarter III

38) If you have been vaccinated, why did you do it (select that apply)?

- To protect myself
- To protect the baby
- Because it was recommended by doctors
- Because it was recommended by the government
- Because it was recommended by the media
- To be able to go on vacation
- To be able to travel for business purposes
- To be able to visit my relatives and/or friends
- To the pressures of the employer
- To be able to access public spaces and institutions where the digital certificate was required

39) If you were vaccinated, did you feel a sense of security and peace as a result?

- Very strong
- Strong
- Moderate
- Weak

- Not at all

40) If you have been vaccinated, have you had any unwanted side effects?

- Minor, local symptoms such as inflammation and pain at the injection site
- Generalized symptoms, affecting the general condition in the short term
- Generalized symptoms with long-term impairment
- Fetal symptoms
- Not

41) If you have not been vaccinated, why not?

- Distrust in manufacturing pharmaceutical companies
- Distrust in government
- Distrust in the one-sided promotional message from the mass media
- Fast-track vaccine design and testing
- Fear of side effects on one's own person
- Fear of side effects on the baby
- Lack of vaccine studies on pregnant women

42) During the pandemic, have you noticed an increase in the frequency of certain habits/behaviors generally associated with stress reduction?

- Not
- Yes, alcohol consumption
- Yes, tobacco smoking
- Yes, using electronic smoking/vaping devices
- Yes, recreational drug use
- Yes, overeating in general
- Yes, excessive eating of sweet/salty/fatty foods
- Yes, others:

43) During the pandemic, have you noticed an increase in conflicts with your partner or other relatives?

- No, and they didn't exist before
- Not
- Yes, a minor increase
- Yes, a noticeable increase
- Yes, a significant increase
